# Twist-tailoring Coulomb correlations in van der Waals homobilayers

**DOI:** 10.1038/s41467-020-16069-z

**Published:** 2020-05-01

**Authors:** Philipp Merkl, Fabian Mooshammer, Samuel Brem, Anna Girnghuber, Kai-Qiang Lin, Leonard Weigl, Marlene Liebich, Chaw-Keong Yong, Roland Gillen, Janina Maultzsch, John M. Lupton, Ermin Malic, Rupert Huber

**Affiliations:** 1 Department of Physics, University of Regensburg, Regensburg, Germany; 2 Department of Physics, Chalmers University of Technology, Gothenburg, Sweden; 3 Institute of Condensed Matter Physics, Friedrich-Alexander University Erlangen-Nürnberg, Erlangen-Nürnberg, Germany

**Keywords:** Two-dimensional materials, Two-dimensional materials, Ultrafast photonics, Nonlinear optics

## Abstract

The recent discovery of artificial phase transitions induced by stacking monolayer materials at magic twist angles represents a paradigm shift for solid state physics. Twist-induced changes of the single-particle band structure have been studied extensively, yet a precise understanding of the underlying Coulomb correlations has remained challenging. Here we reveal in experiment and theory, how the twist angle alone affects the Coulomb-induced internal structure and mutual interactions of excitons. In homobilayers of WSe_2_, we trace the internal 1*s*–2*p* resonance of excitons with phase-locked mid-infrared pulses as a function of the twist angle. Remarkably, the exciton binding energy is renormalized by up to a factor of two, their lifetime exhibits an enhancement by more than an order of magnitude, and the exciton-exciton interaction is widely tunable. Our work opens the possibility of tailoring quasiparticles in search of unexplored phases of matter in a broad range of van der Waals heterostructures.

## Abstract (web summary)

The crystallographic orientation of monolayers in van der Waals multi-layers controls their electronic and optical properties. Here the authors show how the twist angle affects Coulomb correlations governing the internal structure and the mutual interaction of excitons in homobilayers of WSe_2_.

## Introduction

To tackle the formidable many-body scenario of Coulomb correlations in solids, Lev Landau introduced the concept of quasiparticles. These fictitious entities, which consist of particles dressed by their many-body interactions, are usually characteristic of material composition. Yet van der Waals layered materials provide an efficient tuning knob: the twist angle between adjacent layers can turn the semimetal graphene into a Mott insulator^1^, superconductor^2^, or ferromagnet^3^; topological phases^4–7^, spin-pseudospin coupling^8^, and shear solitons^9^ have been discussed for twisted bilayers of transition metal dichalcogenides (TMDs).

TMDs form a unique laboratory to explore such effects because quantum confinement in two dimensions and reduced dielectric screening give rise to exceptionally strong Coulomb interactions. In the monolayer limit, this situation allows hydrogen-like electron-hole pairs—excitons—to bind with energies on the order of hundreds of meV (refs. ^10–12^). In van der Waals heterostructures, the relative orientation between adjacent layers strongly influences the electronic structure because of a subatomic variation of the orbital overlap of adjacent layers^1–6,9,13,14^. Hybridization effects between the electronic states of the constituent layers allow the band structure to be externally controlled by the twist angle *θ* (refs. ^13^^,^^15–20^). Interband photoluminescence combined with theoretical calculations have provided valuable insight into these phenomena^16,21,22^, but cannot resolve the internal excitonic structure which reveals the Coulomb correlations within a bilayer (BL).

Interband spectroscopy can only probe optically bright excitons, whereas the formation and decay dynamics of excitons in prototypical monolayer TMDs such as WSe_2_—and especially in twisted BLs—are often dominated by dark states^23,24^. Mid-infrared (MIR) photons, in contrast, may directly interrogate Lyman-like 1*s*–2*p* transitions of excitonic species, irrespective of interband dipole moments, center-of-mass momenta, spin, or spatial separation of electron and hole. This concept^25^ has been employed to probe Coulomb correlations^26^ in quantum wells^27^, TMD monolayers^28,29^ and heterostructures^30^, where electronic correlations are dominated by chemical composition and quantum confinement.

By exploiting the internal 1*s*–2*p* resonance of excitons in homobilayers of WSe_2_, we demonstrate how the twist angle renormalizes the exciton binding energy by a factor of up to two and enhances their lifetime by more than an order of magnitude, whereas the exciton–exciton interaction is widely tunable at a certain angle. This fascinating scenario can be quantitatively explained with our microscopic many-body theory, which identifies the wavefunction overlap, interlayer hopping, and hybridization effects at the atomic interface as effective means to synthesize custom-tailored hybrid exciton species.

## Results

### Rydberg spectroscopy of excitons in twisted WSe_2_ BLs

**Fig. 1:**
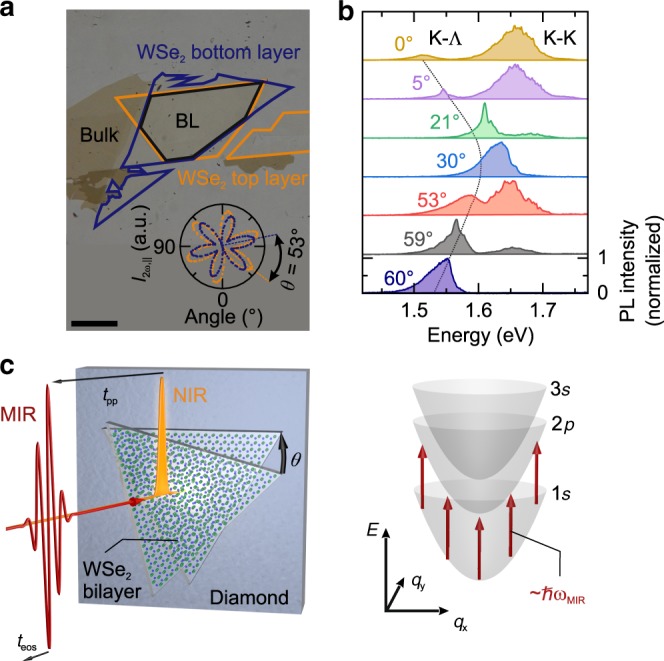
NIR pump–MIR probe spectroscopy of twisted WSe_2_ bilayers. **a** Optical microscope image of a representative twisted homobilayer (BL) on a diamond substrate. The blue frame indicates the lower WSe_2_ monolayer, which is covered by a second WSe_2_ monolayer (orange frame) forming a twisted BL in the overlap region (black frame). Scale bar: 50 µm. Inset: component of the second harmonic *I*_2ω,||_ polarized parallel to the pump laser light. The characteristic patterns of sixfold symmetry indicate the armchair directions of the bottom (blue dots) and top (orange dots) monolayers, enclosing a twist angle, *θ* = 53°, close to the natural 2H configuration. **b** Normalized photoluminescence spectra for samples with different twist angles *θ*, recorded at a temperature of 5 K (vertically offset for clarity). The black dotted line serves as a guide to the eye highlighting the evolution of the K-Λ transition. **c** Sketch of the experiment, where a near-infrared (NIR) pump pulse (orange intensity envelope) optically injects 1*s* A excitons in the WSe_2_ BLs. The excited sample is probed by a mid-infrared waveform (MIR, red curve). The exciton dispersion for a given orbital quantum number is depicted as a function of the center-of-mass momentum *q* (gray paraboloids). Red arrows indicate the 1*s*–2*p* transitions of the exciton, interrogated by the MIR photons at an energy *ħω*_MIR_.

The WSe_2_ BL samples are produced by mechanical exfoliation and subsequent deterministic transfer onto a diamond substrate (see Methods). Monolayer flakes with diameters exceeding 70 µm (Fig. 1a) are stamped on top of each other. Before the transfer, the crystallographic orientations of the layers are determined by polarization-resolved second-harmonic generation^15,31^. The component of the second harmonic *I*_2ω,||_ polarized parallel to the near-infrared pump laser indicates the armchair directions of each monolayer (inset Fig. 1a, blue/orange flower patterns), revealing the relative twist angle *θ* between both layers (see Methods). We fabricated a series of BLs with *θ* ranging from 0° to 60° (see Supplementary Figs. 1 and 2).

The samples were pre-characterized using photoluminescence spectroscopy with an excitation wavelength of 488 nm (see Methods). The resulting spectra (Fig. 1b) feature a single emission peak at an energy of ~1.54 eV for the pristine BL with *θ* = 60° (2H stacking), which we attribute to the phonon-assisted recombination of K-Λ excitons^32^. For *θ* = 59°, this peak blue shifts by 20 meV, whereas a second maximum emerges at an energy of ~1.65 eV, corresponding to the direct K-K transition. This trend continues for *θ* = 53° until the two spectral features merge for *θ* = 30°. When the twist angle is further reduced to *θ* = 5° and *θ* = 0° (i.e., the 3R stacking) the two peaks are discernible again and move towards energies of 1.52 eV and 1.66 eV, respectively. Similar photoluminescence characteristics of BLs have been reported in the literature^16,17,21^.

To explore the actual microscopic origin and the role of Coulomb correlations in the photoexcited twisted BLs, we study the internal structure and binding energy of the excitons. To this end, we generate 1*s* A excitons in the WSe_2_ BLs with a 100-fs near-infrared (NIR) laser pulse centered at an energy of 1.68 eV (Fig. 1c, orange intensity envelope). After a variable pump-probe delay time *t*_pp_, the exciton ensemble is probed by a phase-locked MIR pulse (Fig. 1c, red wave), the electric field of which is monitored by electro-optic sampling (see Methods and Supplementary Fig. 3). The intraexcitonic 1*s*–2*p* transition (Fig. 1c, red arrows) imprints a characteristic change on the MIR waveform, from which information about the excitonic correlations of the non-equilibrium system can be extracted. A Fourier domain Fresnel analysis^26–28,30^ directly reveals the pump-induced change of the full dielectric response, including the change of the real part of the optical conductivity, Δ*σ*_1_, and the dielectric function, Δ*ε*_1_, at variable delays *t*_pp_.

**Fig. 2:**
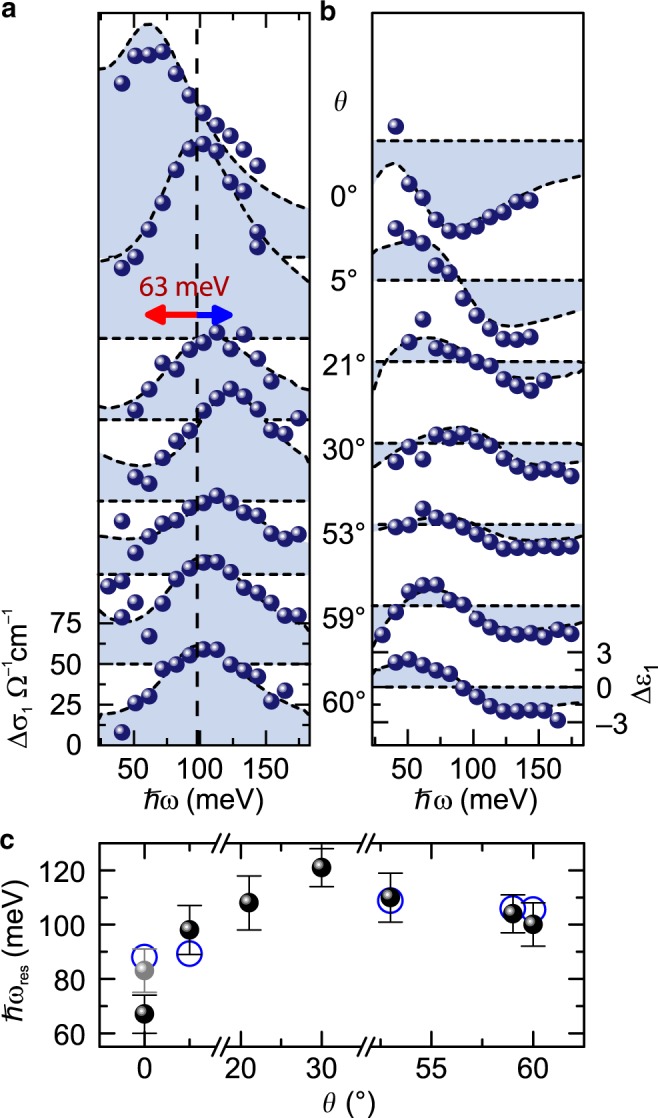
Evolution of the 1*s*–2*p* resonance with interlayer twist angle. **a**, **b** Pump-induced changes of the real parts of the optical conductivity Δ*σ*_1_ (**a**) and the dielectric function Δ*ε*_1_ (**b**) for a fixed pump-probe delay time *t*_pp_ = 5.1 ps as a function of the photon energy for samples with different twist angles *θ*. The blue spheres represent the dielectric response measured for the photoexcited WSe_2_ BLs (pump fluence *Φ* = 27 µJ cm^−2^; sample temperature, 5 K). The blue shaded areas indicate the fits to the experimental data by a phenomenological model (see Methods). The dashed line and the blue/red arrow indicate the blue/red shift of the intraexcitonic 1*s*–2*p* resonance energy for increasing misalignment angle with respect to the 2H stacking. **c** Intraexcitonic 1*s*–2*p* transition energy *ħω*_res_ (black spheres) extracted from the data in **a**, **b** and derived from the microscopic theory (blue circles) as function of the twist angle *θ*. For *θ* = 21° and *θ* = 30°, microscopic calculations of hybrid excitons were not feasible. In the case of *θ* = 0°, the intraexcitonic resonance exhibits a strong dependence on the exciton density (see Fig. 4c and Supplementary Note 4). The gray sphere indicates the resonance energy as obtained for a pump fluence of *Φ* = 7 µJ cm^−2^, yielding the best agreement with the microscopic theory. The error bars represent the 95% confidence interval of the fitting procedure.

Fig. 2 summarizes the changes in dielectric function measured for seven WSe_2_ BLs with varying twist angles *θ*, at *t*_pp_ = 5.1 ps, where the photoexcited state has thermalized (see Supplementary Fig. 4). For all twist angles, Δ*σ*_1_ (Fig. 2a) exhibits a distinct maximum with a corresponding dispersive feature in Δ*ε*_1_ (Fig. 2b), signifying a resonant response of the photoexcited sample. As we demonstrate below, these resonances relate to the internal 1*s*–2*p* transition of BL excitons. Between *θ* = 60° and 30°, the resonance blue shifts with decreasing angle (Fig. 2a dashed line, blue arrow). Strikingly, for *θ* = 0° the resonance exhibits the opposite behavior: it shifts substantially to the red and gains oscillator strength (red arrow). A phenomenological analysis of the data with a two-component model (see Methods) confirms a systematic blue shift of the resonance (Fig. 2c) from 100 meV for *θ* = 60° to 121 meV for *θ* = 30°. Importantly, the resonance at *θ* = 0° is red shifted to 67 meV—corresponding to an energy reduction by a factor of two. This strong asymmetry of the 1*s*–2*p* transition energy—a direct measure of the exciton binding energy^28,30^ (see Methods)—for *θ* = 0° and *θ* = 60° is in stark contrast to the almost degenerate interband photoluminescence (see Fig. 1b).

### Microscopic many-body theory

**Fig. 3:**
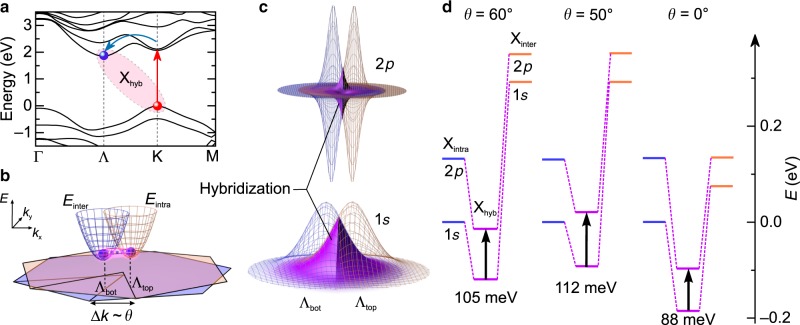
Hybridization between intra- and interlayer excitons. **a** Single-particle band structure of the WSe_2_ BLs for *θ* = 60°, calculated by density functional theory (see Methods). After resonant excitation (red arrow) at the K point, electrons scatter via phonons (blue arrow) into the energetically favorable Λ valley to form hybrid excitons (X_hyb_). **b** Within the microscopic model, the electron is located in a Λ-like valley of the top (Λ_top_) or bottom (Λ_bot_) layer for a finite crystallographic misalignment. The parabolic dispersion of intra- (*E*_intra_) and interlayer (*E*_inter_) excitons is shifted in momentum space by twisting the layers, owing to the momentum mismatch Δ*k* between Λ_top_ and Λ_bot_. Strong interlayer wavefunction overlap promotes electron hopping and exciton hybridization (pink glow). **c** The overlap (violet surface) of excitonic wavefunctions (blue and orange grids) in momentum space depends on Δ*k*. For clarity, we only show the real part of the exciton wavefunctions. **d** Hybridization of 1*s* and 2*p* excitons lowers the energy levels (magenta lines) with respect to the corresponding non-hybridized states X_intra_ and X_inter_ (blue/orange lines). The situation is sketched for *θ* = 60°, *θ* = 50°, and *θ* = 0°. Zero energy is set to the non-hybridized 1*s* state at *θ* = 60°. The vertical black arrows mark respective 1*s*–2*p* transition energies.

To explain these surprising observations, we have to go beyond single-particle band structure effects and include Coulomb correlations, by a combination of ab initio density functional theory (DFT) calculations with density matrix theory (see Methods). In WSe_2_ BLs, the energetic minimum of the conduction band lies at the Λ and Λ′ points. Thus, following creation of excitons at the Κ point (Fig. 3a, red arrow), electrons scatter via phonons into the global energetic minima^33^ (Fig. 3a, blue arrow), corroborated by a transient shift of the excitonic 1*s*–2*p* resonance (see Supplementary Fig. 4). The conduction band states in the Λ and Λ′ valleys are composed of selenium *p* orbitals, whose overlap with the corresponding wavefunctions of the neighboring layer leads to efficient interlayer hopping^34^ and hybridization of the excitonic wavefunctions (X_hyb_). We study this scenario by tracing excitons with their hole fixed in one layer—without loss of generality assigned to the top layer—whereas the electrons are located in Λ-like valleys of either the top (Λ_top_) or the bottom (Λ_bot_) layer (see Fig. 3b and Methods), forming intra- (X_intra_) or interlayer excitons (X_inter_). The energy of both exciton species is influenced by the electron-hole Coulomb attraction. The interlayer exciton energy depends further on the spin-orbit splitting of the conduction band in the bottom layer. The parabolic dispersion relations of the excitons, *E*_intra_ and *E*_inter_, are mutually offset in momentum space by *θ* (see Fig. 3b). Depending on the wavefunction overlap of X_intra_ and X_inter_ in momentum space (Fig. 3c, blue/orange grid), these states can hybridize effectively. The overlap (Fig. 3c, violet surfaces) depends on *θ* and the orbital quantum number of the excitonic states, which defines the momentum spread of the wavefunctions. Upon hybridization, bonding and anti-bonding exciton states are formed. As the energy splitting between bonding and anti-bonding 1*s* states is as large as ~500 meV, the anti-bonding exciton state is pushed outside of our MIR probe spectrum. Therefore, we only consider the energetically lower 1*s*- and 2*p*-hybrid states (Fig. 3d, magenta lines). As the energy reduction differs for these two states, the 1*s*–2*p* transition energy varies with *θ* (Fig. 3d).

For *θ* = 60°, X_intra_ and X_inter_ feature very different energies (Fig. 3d). This situation is characteristic of the 2H order, where the momentum overlap is strongest for intra- and interlayer exciton states containing electrons from the Λ and Λ′ valley, respectively. As hybridization can only occur for spin-like electron bands and spin-orbit splitting is of opposite sign for the two valleys, the non-hybridized excitons are offset by the strong spin-orbit splitting. Despite this energy mismatch, the energy of X_hyb_ is clearly reduced. For increasing misalignment (*θ* < 60°) with respect to 2H stacking, the 1*s* states, which are spread out over a broad range of momenta, continue to effectively hybridize. Conversely, the 2*p* wavefunctions are more strongly localized in momentum space. Hence misalignment towards *θ* = 30° rapidly quenches hybridization in the 2*p* state and therefore reduces the energy gain. The combination of these mechanisms results in a distinct blue shift of the 1*s*–2*p* resonance, in excellent agreement with the experiment (Fig. 2c, blue circles). For 3R stacking, X_intra_ and X_inter_ are almost degenerate because both states contain electrons from the same valley and are therefore not offset by spin-orbit splitting. In particular, the 2*p* states are almost perfectly degenerate, which enhances the excitonic hybridization as compared to the pristine bilayer (*θ* = 60°) and leads to an even smaller 1*s*–2*p* transition energy (see Fig. 3d).

### Tailoring the excitonic lifetime

**Fig. 4:**
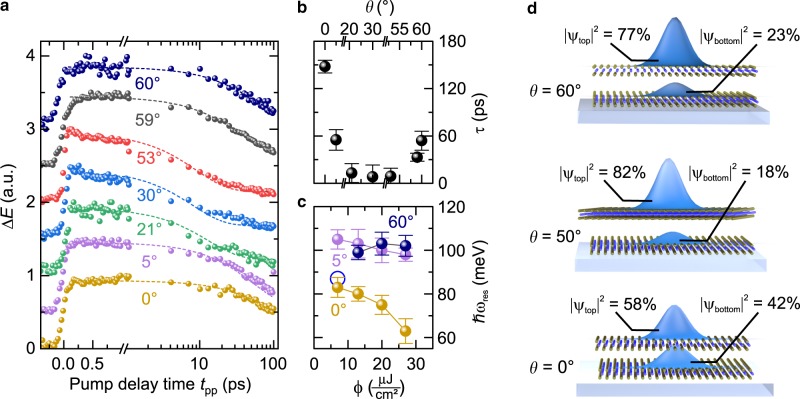
Ultrafast dynamics and density-dependent energy renormalization of hybridized excitons. **a** Pump-induced change of the mid-infrared electric field Δ*E* as a function of the pump-probe delay time *t*_pp_ recorded at fixed electro-optic sampling time *t*_eos_ = 0 fs for different twist angles *θ* (spheres). Curves are vertically offset for clarity. The dashed lines represent fits to the data with an exponential decay. **b** Exciton decay time *τ* as a function of the twist angle *θ*, determined by the decay constant of the fit in **a**. The error bars indicate one standard deviation. **c** 1*s*–2*p* resonance energy *ħω*_res_ for different pump fluences *Φ* at a pump-probe delay time *t*_pp_ = 5.1 ps recorded for twist angles *θ* = 0°, 5°, and 60°, respectively (spheres). The resonance energies were extracted from the dielectric response by fits to a phenomenological model (see Supplementary Fig. 5). The blue circle marks the resonance energy derived from the microscopic theory shown in Fig. 2c. The sample was kept at a temperature of 5 K. The error bars represent the 95% confidence interval of the fitting procedure. **d** Calculated probability density of the electron within the two layers for a hybrid exciton for twist angles *θ* = 60°, 50°, and 0°, where the hole is chosen to remain at a fixed position in the top layer.

The variable degree of interlayer hybridization of excitons allows us to tune the exciton dynamics as well as the exciton–exciton interaction (Fig. 4). We trace the temporal evolution of the exciton population by mapping out the pump-induced change of the MIR electric field Δ*E* at a fixed electro-optic sampling time, *t*_eos_ = 0 fs (see ref. ^28^ and Methods), for a variable pump-probe delay time *t*_pp_. Fig. 4a shows a rapid appearance of exciton density at *t*_pp_ = 0 ps, for all twist angles, whereas the decay dynamics depend sensitively on *θ*. An exponential fit (Fig. 4a, dashed lines) shows a systematic reduction of the lifetime of the hybrid excitons (Fig. 4b) from *τ* = 54 ps to 8 ps as *θ* is reduced from 60° to 30°. Intriguingly, for *θ* = 0°, the lifetime rises dramatically to a value of 148 ps. This behavior can be understood by considering the hybridized wavefunctions in real space.

To do this, we keep the position of the hole fixed in the upper TMD layer and plot the probability density of the electron (Fig. 4d, blue peaks). Because of strong interlayer hopping between the Λ and Λ′ points of the adjacent layers, the electron delocalizes within the BL for *θ* = 60°. A distinct probability density in the bottom layer (23%) signifies the interlayer character of the hybrid exciton. By changing the twist angle to *θ* = 50°, the orbital overlap of the conduction band states shrinks, reducing the probability density of the electron in the bottom layer (18%). With both electron and hole wavefunctions overlapping strongly in the top layer, the lifetime of the exciton drops, as processes such as phonon-assisted radiative decay^32,35^ or Auger recombination^28^ likely become more efficient. For *θ* = 30°, the interlayer hopping becomes even weaker, which further reduces the exciton lifetime. In contrast, for *θ* = 0° interlayer hopping is facilitated by the energetic degeneracy of the Λ-valley conduction band states discussed above. In this case, the strong delocalization of the electron over the top (58%) and bottom (42%) layers distinctly extends the exciton lifetime as the hybrid state becomes more interlayer-like. At the same time, the delocalized wavefunctions provide a larger transition dipole moment, enhancing the amplitude of Δ*σ*_1_ for *θ* = 0° as compared with *θ* = 60° (Fig. 2a).

### Shaping the interaction between excitons

The twist angle controlled interlayer delocalization can also be employed to tune the interaction between excitons. To this end, we record the position of the 1*s*–2*p* resonance as a function of the pump fluence (Fig. 4c). For *θ* = 0°, a dramatic red shift is observed, whereas the effect is less pronounced for *θ* = 5° and completely absent for *θ* = 60° (see Supplementary Fig. 5, for the full dielectric response). We expect that an effective renormalization of the exciton binding energy should ultimately occur for all twist angles, when the exciton densities are increased beyond the values accessible in our experiment. Microscopically, the critical exciton density at which screening effects become important is determined by the exciton wavefunction. The larger the respective Bohr radius, the lower the critical density at which the orbitals start to overlap. Hence, strong interlayer delocalization accompanied by a larger lateral extent of the wavefunction (*θ* = 0°, see Fig. 4d) enables excitons to effectively screen the mutual Coulomb attraction between electron and hole. In addition, the delocalization of the excitons across the two layers for *θ* = 0° increases their polarizability and enables an out-of-plane dipole-dipole type interaction^36^. Consequently, hybrid excitons experience a stronger renormalization of their internal transitions with increasing exciton density as compared to previous reports on intralayer excitons^28,37^. Thus, for increasing pump fluences the exciton binding energy is further reduced beyond the value of the hybrid exciton with negligible many-body interactions. Therefore, the theoretically predicted transition energy (see Figs. 2c and 4c, blue circle) is in very good agreement with the experimental results only for low fluences (compare grey sphere in Fig. 2c).

## Discussion

In conclusion, we have tailored Coulomb correlations by interlayer hybridization in twisted WSe_2_ BLs. By accessing internal 1*s*–2*p* transitions of the K-Λ excitons, we reveal systematic changes of their binding energy and lifetime with twist angle. A microscopic model based on density functional and density matrix theory quantitatively describes the underlying hybridization effects. Furthermore, we reveal how the density-dependent exciton–exciton interaction is renormalized by the twist angle. These findings underpin a qualitatively different way of fine-tuning the internal structure of excitons in TMDs and controlling the electronic and optical properties in van der Waals structures based on a single material. Tailoring many-body correlations over a large energy range can now be systematically extended to the full library of two-dimensional crystals, which should guide the search for man-made electronic phases in van der Waals heterostructures.

## Methods

### Sample fabrication and photoluminescence spectroscopy

The TMD monolayers were exfoliated mechanically from a bulk single crystal using the viscoelastic transfer method^38^. Both constituent WSe_2_ monolayers were inspected under an optical microscope and subsequently stacked on top of each other on a diamond substrate. During the transfer process, the lateral position and rotation of the layers can be controlled accurately using micro-positioning stages. To remove any adsorbates trapped between the monolayers, the BLs were annealed at a temperature of 150 °C and a pressure of 1 × 10^−5^ mbar for 5 hours. Subsequently, we recorded the microphotoluminescence intensity of the BLs with a continuous-wave laser (wavelength, 488 nm) focused onto the sample using a microscope objective (numerical aperture, 0.6). The radiation emitted by the BLs is then detected in backscattering geometry, dispersed in a spectrometer, and read out by a charge-coupled device camera.

### Second-harmonic generation

The twist angle *θ* was determined for each BL by polarization-resolved second-harmonic generation (SHG) under rotation of the plane of polarization^31^. Isolated monolayer regions of the structure were excited by a linearly-polarized femtosecond mode-locked Ti:sapphire laser and the intensity of the SHG was recorded. Characteristic sixfold patterns, whose maxima indicate the armchair directions of the monolayers, are then obtained. For the representative structure in Fig. 1a, the intensity maxima span an angle ~7°. As the method is not sensitive to the electric field of the second harmonic but only to its intensity, an ambiguity remains between twist angles of *θ* = 7° and *θ* = 60°-7°. This ambiguity can be resolved by recording the sixfold pattern of the SHG polarization anisotropy of the bilayer area of the sample (see Supplementary Fig. 2). The strong suppression of the SHG emission of the bilayer region attests to the fact that the two layers are almost perfectly anti-aligned^39,40^ corresponding to an angle close to the natural 2H stacking. Thus, the twist angle of the sample presented in Fig. 1a can be determined as *θ* = 53°. For the other BLs, an identical procedure is performed (see Supplementary Note 1).

### Ultrafast NIR pump–MIR probe spectroscopy

A schematic of the experimental principle is depicted in Fig. 1c. 1*s *A excitons are resonantly created in the WSe_2_ BL by photoexcitation with 100-fs pump pulses centered at an energy of 1.69 eV (Supplementary Fig. 3a, inset) from a home-built Ti:sapphire laser amplifier (repetition rate, 400 kHz). The phase-locked MIR probe pulses are generated by optical rectification in a GaSe crystal (thickness, 10 µm) using a fraction of the laser output. After a variable delay time *t*_pp_, the probe pulses are transmitted through the photoexcited sample and the MIR waveform is recorded by electro-optic sampling in a second 10 µm-thick GaSe crystal as a function of the electro-optic sampling time *t*_eos_ (Supplementary Fig. 3a). The MIR probe pulses are centered at a frequency of 28 THz with a full width at half maximum of 14 THz and a spectral phase, which is approximately flat between 11 and 44 THz (Supplementary Fig. 3b). Any changes to the electric field, Δ*E*(*t*_eos_), of the waveform induced by the transient dielectric response of the sample are resolved in absolute amplitude and phase by serial lock-in detection as a function of the electro-optic sampling time *t*_eos_. The full complex-valued dielectric response, characterized by Δ*σ*_1_ and Δ*ε*_1_, can be retrieved from these data by applying a transfer matrix formalism^26,28,30^. This technique is sensitive to the total population of bound and unbound electron-hole pairs, irrespective of interband selection rules, and can, thus, address optically dark and bright excitons^41^.

### Microscopic many-particle theory

To calculate the internal quantum transitions of excitons in the twisted BL, a many-particle Hamiltonian was diagonalized accounting for the Coulomb interaction on a Hartree-Fock level. For WSe_2_ BLs, ab initio calculations suggest that momentum-dark K-Λ excitons represent the lowest states. Therefore, the Hamiltonian was restricted to electrons and holes at these high-symmetry points, taking into account the energetically most favorable dipole-allowed spin configuration. The BL was then treated as a bipartite system, using the monolayer eigenstates as basis functions of the two subsystems. Here, the layer-localized basis functions were treated in an effective mass approximation with parameters extracted from ab initio simulations for monolayers. The Coulomb interaction strength was implemented on a semiclassical level assuming anisotropic homogeneous slabs to account for the complex dielectric environment of the BL system. Ab initio parameters for the anisotropic dielectric tensor of WSe_2_ were used and the influence of the substrate was included. The overlap of electronic wavefunctions of the two-layer subsystem gives rise to interlayer hybridization effects. Only the hybridization of electrons at the Λ point was considered, which is known to be orders of magnitude stronger than at the K point owing to different orbital compositions. The interlayer interaction strength was extracted from ab initio calculations of the BL band structure for different stacking angles. The Hamiltonian was diagonalized by first transforming it into an exciton frame to account for the Coulomb interaction. Subsequently, eigenenergies and wavefunctions were represented in terms of intra- and interlayer excitons, resulting from the Wannier equation for the BL. The overlap of electronic wavefunctions between the two layers results in an interaction of intra- and interlayer excitons. Finally, the excitonic Hamiltonian was diagonalized yielding intralayer-interlayer exciton hybrids. For the numerical calculation of the bilayer eigenstates we consider the hybridization of a large number of both intra- and interlayer excitons including states with angular momentum of *s-*, *p-*, *d-,* and *f*-type. The measured transition energies were then compared with the energetically lowest dipole-allowed transition of the calculated hybrid exciton Rydberg series where we neglect higher-order corrections such as a Berry curvature-induced splitting of the otherwise degenerate *p* states. For additional details see Supplementary Note 5.

### Ab initio DFT calculations

The electronic band structure was calculated on the basis of density functional theory in the Perdew-Burke-Ernzerhof approximation, as implemented in the Quantum ESPRESSO package. The atomic positions and in-plane lattice constants of twisted BL WSe_2_ were optimized with different relative twist angles and the resulting atomic geometries were used to derive an average equilibrium distance between the two tungsten sublayers. Based on the optimized geometry of a 2H stacked BL, the evolution of the electronic band structure for varying interlayer distance was simulated. The required interlayer interaction strength to calculate the exciton hybridization was extracted from the splitting of the electronic bands at the Λ point. To obtain a continuous twist angle dependence of the interaction parameter, the interlayer distances obtained for computationally feasible angles were linearly interpolated. Spin-orbit interactions and quasiparticle energy corrections from the G_0_W_0_ approximation were explicitly included to calculate the full band structure for 2H stacked WSe_2_. Here, the YAMBO code was used to calculate the quasiparticle corrections with spin-orbit effects. Subsequently, GW calculations with the BerkeleyGW code were performed, combined with a non-uniform Neck Subsampling method to derive an additional scissor-shift correction for the electronic band gaps. For additional details, see Supplementary Note 6.

### Two-component model

A phenomenological two-component model was used to extract the quantitative properties of the 1*s*–2*p* transition from the transient dielectric response. To this end, the pump-induced change of the dielectric function Δ*ε*(*ω*)  = Δ*ε*_1_(*ω*) + Δ*σ*_1_(*ω*)/(*ε*_0_*ω*) is described by the Drude–Lorentz model:

1 $$\Delta \varepsilon \left( \omega \right) = \frac{{n_{\mathrm{X}}e^2}}{{d\varepsilon _0\mu }}\frac{{f_{1s,2p}}}{{\omega _{{\mathrm{res}}}^2 - \omega ^2 - {\mathrm{i}}\omega {\mathrm{\Delta }}}} - \frac{{n_{{\mathrm{fc}}}e^2}}{{d\varepsilon _0{\upmu}}}\frac{1}{{\omega ^2 + {\mathrm{i}}\omega \Gamma }}$$

where *e* is the electron charge and *ε*_0_ denotes the vacuum permittivity. The Lo-rentzian resonance with oscillator strength *f*_1*s*,2*p*_ describes the internal 1*s*–2*p* transition of the excitons within the WSe_2_ BL as determined by the density matrix theory (see Methods). Owing to the twist angle-dependent hybridization, the oscillator strength equals *f*_1*s*,2*p*_ = 0.34, *f*_1*s*,2*p*_ = 0.33, *f*_1*s*,2*p*_ = 0.23, *f*_1*s*,2*p*_ = 0.18, *f*_1s,2p_ = 0.19, *f*_1*s*,2*p*_ = 0.26, and *f*_1*s*,2*p*_ = 0.42 for *θ* = 60°, 59°, 53°, 30°, 21°, 5°, and 0°, respectively. The resonance is further characterized by the exciton density *n*_X_, the resonance energy *ħω*_res_ and the linewidth Δ, which are free fit parameters. The effective mass of the electron-hole pairs *μ* = 0.23 *m*_0_ is determined with the electron and hole effective masses within the WSe_2_ BL (ref. ^42^), where *m*_0_ is the free-electron mass. The sample is treated as a thin slab in this model with a thickness *d* = 0.7 nm (ref. ^28^) of each layer. The second term in Δ*ε*(*ω*) is a Drude model describing unbound electron-hole pairs or excitonic transitions energetically below the spectral window of the MIR probe pulses^30^. Here, the density of free carriers *n*_fc_ and their scattering rate Γ govern the resulting dielectric response. Strict limits on the possible values of the free parameters are set by the fact that both independently retrieved spectra Δ*σ*_1_ and Δ*ε*_1_ have to be reproduced simultaneously. Without any further restrictions, the numerical adaption of the measured spectra yields an overall good fit quality.

## Supplementary information

Supplementary Information

## Data Availability

The data sets generated during and/or analyzed during the current study are available from the corresponding author on reasonable request.

## References

[1] Cao Y (2018). Correlated insulator behaviour at half-filling in magic-angle graphene superlattices. Nature.

[2] Cao Y (2018). Unconventional superconductivity in magic-angle graphene superlattices. Nature.

[3] Sharpe AL (2019). Emergent ferromagnetism near three-quarters filling in twisted bilayer graphene. Science.

[4] Tong Q (2016). Topological mosaics in moiré superlattices of van der Waals heterobilayers. Nat. Phys..

[5] Wu F, Lovorn T, MacDonald AH (2017). Topological exciton bands in Moiré Heterojunctions. Phys. Rev. Lett..

[6] Yu H, Liu G-B, Tang J, Xu X, Yao W (2017). Moiré excitons: from programmable quantum emitter arrays to spin-orbit–coupled artificial lattices. Sci. Adv..

[7] Wu F, Lovorn T, Tutuc E, Martin I, MacDonald AH (2019). Topological insulators in twisted transition metal dichalcogenide homobilayers. Phys. Rev. Lett..

[8] Jin C (2019). Identification of spin, valley and moiré quasi-angular momentum of interlayer excitons. Nat. Phys..

[9] Naik MH, Jain M (2018). Ultraflatbands and shear solitons in Moiré patterns of twisted bilayer transition metal dichalcogenides. Phys. Rev. Lett..

[10] Novoselov KS, Mishchenko A, Carvalho A, Castro Neto AH (2016). 2D materials and van der Waals heterostructures. Science.

[11] Ugeda MM (2014). Giant bandgap renormalization and excitonic effects in a monolayer transition metal dichalcogenide semiconductor. Nat. Mater..

[12] Xu X, Yao W, Xiao D, Heinz TF (2014). Spin and pseudospins in layered transition metal dichalcogenides. Nat. Phys..

[13] Alexeev EM (2019). Resonantly hybridized excitons in moiré superlattices in van der Waals heterostructures. Nature.

[14] Wang, L. et al. Magic continuum in twisted bilayer WSe_2_. Preprint at https://arxiv.org/abs/1910.12147 (2019).

[15] Kunstmann J (2018). Momentum-space indirect interlayer excitons in transition-metal dichalcogenide van der Waals heterostructures. Nat. Phys..

[16] Van Der Zande AM (2014). Tailoring the electronic structure in bilayer molybdenum disulfide via interlayer twist. Nano Lett..

[17] Jiang T (2014). Valley and band structure engineering of folded MoS_2_ bilayers. Nat. Nanotechnol..

[18] Horng J (2018). Observation of interlayer excitons in MoSe_2_ single crystals. Phys. Rev. B.

[19] Hsu W-T (2019). Tailoring excitonic states of van der Waals bilayers through stacking configuration, band alignment, and valley spin. Sci. Adv..

[20] Gerber IC (2019). Interlayer excitons in bilayer MoS_2_ with strong oscillator strength up to room temperature. Phys. Rev. B.

[21] Liu K (2014). Evolution of interlayer coupling in twisted molybdenum disulfide bilayers. Nat. Commun..

[22] Wang Z, Chiu Y-H, Honz K, Mak KF, Shan J (2018). Electrical tuning of interlayer exciton gases in WSe_2_ bilayers. Nano Lett..

[23] Nayak PK (2017). Probing evolution of twist-angle-dependent Interlayer excitons in MoSe_2_/WSe_2_ van der Waals heterostructures. ACS Nano.

[24] Zhang X-X, You Y, Zhao SYF, Heinz TF (2015). Experimental evidence for dark excitons in monolayer WSe_2_. Phys. Rev. Lett..

[25] Ulbricht R, Hendry E, Shan J, Heinz TF, Bonn M (2011). Carrier dynamics in semiconductors studied with time-resolved terahertz spectroscopy. Rev. Mod. Phys..

[26] Huber R (2001). How many-particle interactions develop after ultrafast excitation of an electron–hole plasma. Nature.

[27] Kaindl RA, Carnahan MA, Hägele D, Lövenich R, Chemla DS (2003). Ultrafast terahertz probes of transient conducting and insulating phases in an electron–hole gas. Nature.

[28] Poellmann C (2015). Resonant internal quantum transitions and femtosecond radiative decay of excitons in monolayer WSe_2_. Nat. Mater..

[29] Yong C-K (2019). Valley-dependent exciton fine structure and Autler–Townes doublets from Berry phases in monolayer MoSe_2_. Nat. Mater..

[30] Merkl P (2019). Ultrafast transition between exciton phases in van der Waals heterostructures. Nat. Mater..

[31] Lin K-Q, Bange S, Lupton JM (2019). Quantum interference in second-harmonic generation from monolayer WSe_2_. Nat. Phys..

[32] Lindlau J (2018). The role of momentum-dark excitons in the elementary optical response of bilayer WSe_2_. Nat. Commun..

[33] Waldecker L (2017). Momentum-resolved view of electron-phonon coupling in multilayer WSe_2_. Phys. Rev. Lett..

[34] Liu G-B, Xiao D, Yao Y, Xu X, Yao W (2015). Electronic structures and theoretical modelling of two-dimensional group-VIB transition metal dichalcogenides. Chem. Soc. Rev..

[35] Brem S (2020). Phonon-assisted photoluminescence from indirect excitons in monolayers of transition-metal dichalcogenides. Nano Lett..

[36] Miller B (2017). Long-lived direct and indirect interlayer excitons in van der waals heterostructures. Nano Lett..

[37] Cunningham PD, Hanbicki AT, McCreary KM, Jonker BT (2017). Photoinduced bandgap renormalization and exciton binding energy reduction in WS_2_. ACS Nano.

[38] Castellanos-Gomez A (2014). Deterministic transfer of two-dimensional materials by all-dry viscoelastic stamping. 2D Mater..

[39] Leandro M (2014). Observation of intense second harmonic generation from MoS_2_ atomic crystals. Phys. Rev. B.

[40] Hsu W (2014). Second harmonic generation from artificially stacked transition metal dichalcogenide twisted Bilayers. ACS Nano.

[41] Berghäuser G (2018). Mapping of the dark exciton landscape in transition metal dichalcogenides. Phys. Rev. B.

[42] Kormányos A (2015). k·p theory for two-dimensional transition metal dichalcogenide semiconductors. 2D Mater..

