# Supplementary material for: Twist-tailoring Coulomb correlations in van der Waals homobilayers
Source: Nat Commun. 2020 May 1;11:2167. doi: 10.1038/s41467-020-16069-z (PMC7195450; doi:10.1038/s41467-020-16069-z)
Supplement: Supplementary file 1 — Supplementary Information [file 41467_2020_16069_MOESM1_ESM.pdf]

Supplementary Information for

Twist-tailoring Coulomb correlations in van der Waals homobilayers

Merkl *et al.*

Supplementary Note 1

The sample fabrication is detailed in the Methods section of the manuscript. Optical microscope images of the resulting structures are shown in Supplementary Fig. 1. For each sample, the two constituent monolayers form a homobilayer (BL) region exceeding $60 \times 60\ \mu m^2$ in size. The relative orientation of the WSe_2 monolayers covers the whole range of twist angles θ from 0° to 60°.

The crystallographic orientation of the monolayers was determined by polarization-resolved second-harmonic generation as outlined in the Methods section. Supplementary Fig. 2 depicts the resulting six-fold patterns of the intensity component polarized parallel to the laser fundamental, $I_{2\omega,\|}$. The maxima of these patterns indicate the armchair directions of the bottom monolayer (blue) and the top monolayer (orange), which were accurately extracted by a fitting procedure using $\cos^2(3\beta-\gamma)$, where β indicates the orientation of the linearly polarized fundamental and γ the relative orientation of the armchair direction[1]. In Supplementary Fig. 2a, the data from Fig. 1a of the main manuscript is reproduced. Additionally, the intensity $I_{2\omega,\|}$ recorded on the BL region is depicted. The strong destructive interference attests to an alignment close to the natural 2H configuration confirming a twist angle of $\theta = 53°$ for this sample. Similarly, Supplementary Fig. 2b shows the second harmonic intensity $I_{2\omega,\|}$ recorded on another sample. Due to the strong destructive interference of the second harmonic generated from the constituent monolayers of the BL region, a twist angle of $\theta = 59°$ is extracted in this case. For the sample shown in Supplementary Fig. 2c, we determine a twist angle of $\theta = 21°$ owing to the constructive interference occurring in second-harmonic generation.

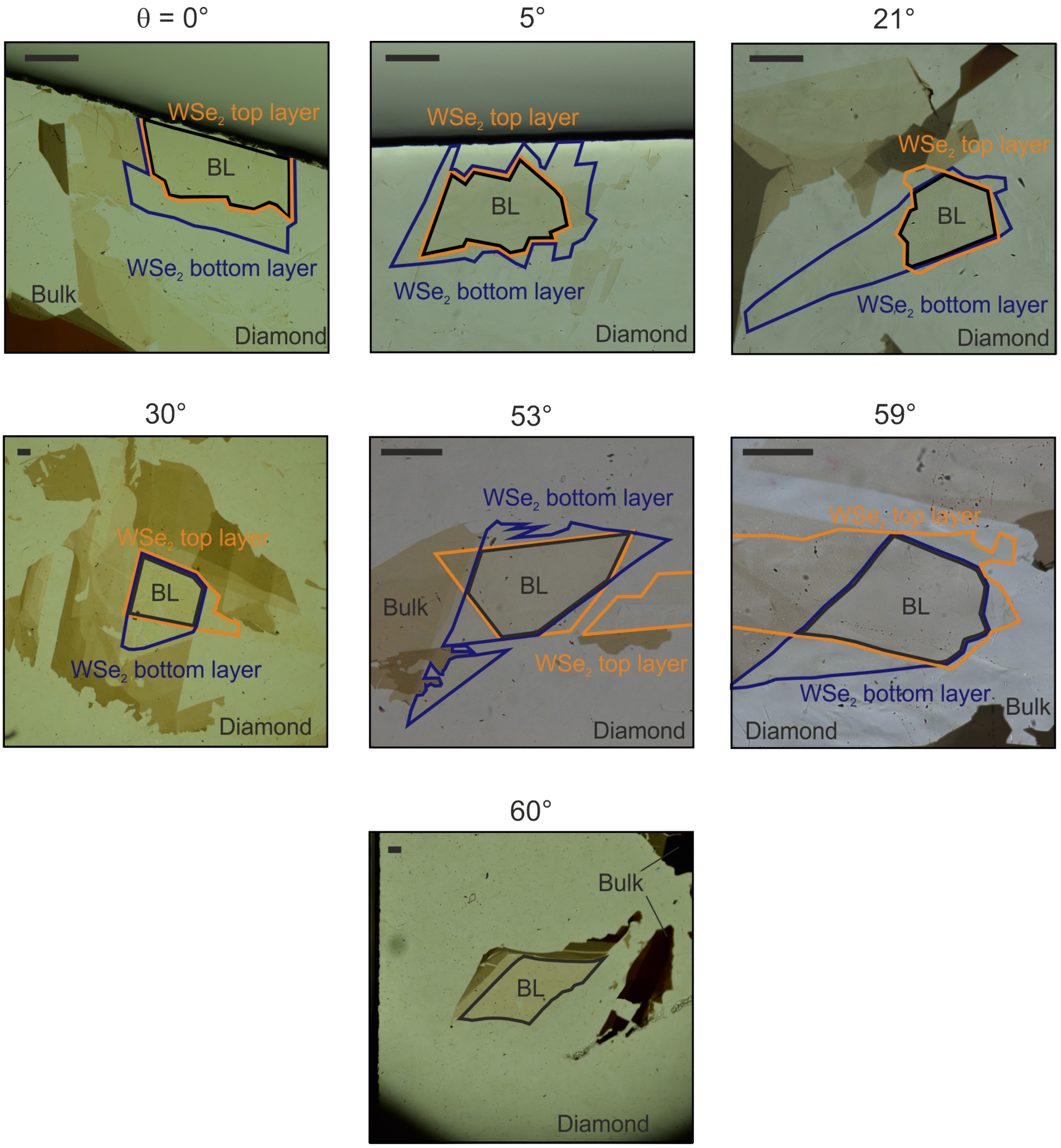

Supplementary Figure 1 | Overview of the homobilayer samples. Optical microscope images of the BLs with twist angles $\theta = 0°$, 5°, 21°, 30°, 53°, 59° and 60° on diamond substrates. The blue frames indicate the bottom WSe_2 monolayers, which were transferred first. Each of the monolayers is covered by an additional top WSe_2 monolayer outlined by the orange frame thereby forming a BL indicated by the black frame. Scale bars: 50 µm.

For some of the samples in Supplementary Fig. 1, the twist angle could be determined in an alternative way. For example, the structure with a twist angle of $\theta = 60°$ is a pristine, as-exfoliated BL. The remaining samples were produced starting from an extremely large exfoliated monolayer. Then, only roughly half of this monolayer was transferred onto the substrate. This was achieved by rapidly retracting the transfer stage from the substrate during the transfer process, effectively splitting the monolayer into two pieces. Thus, the part of the monolayer, which remained on the transfer substrate, was perfectly aligned with the monolayer on the diamond substrate. By a precise rotation of the diamond substrate, the twist angle could then be controlled *in situ* without resorting to polarization-resolved second-harmonic generation.

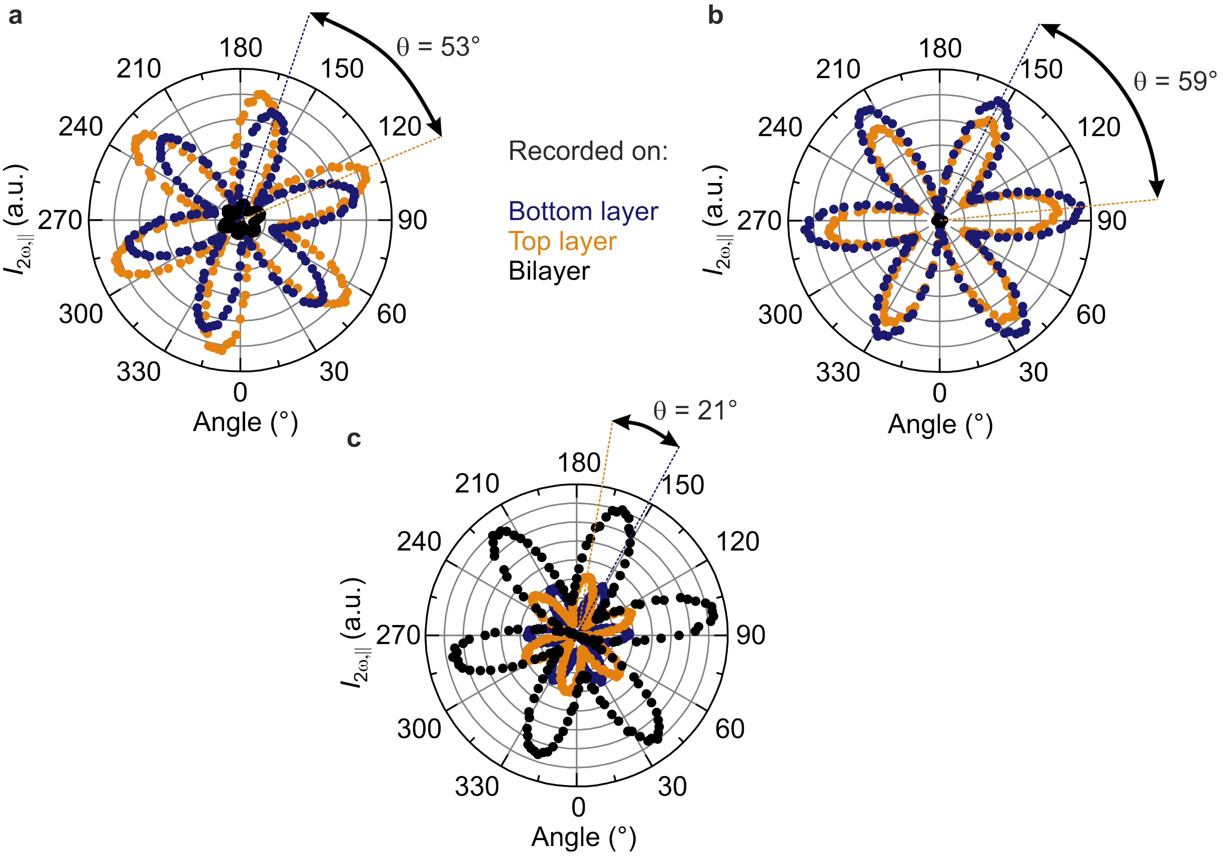

Supplementary Figure 2 | Determination of the twist angle by second harmonic generation. Component of the second-harmonic intensity $I_{2\omega,\parallel}$ polarized parallel with respect to the linear polarization of the laser excitation. The characteristic six-fold patterns indicate the armchair directions of the bottom (blue dots) and top (orange dots) monolayers. Additionally, the second-harmonic intensity recorded on the BL region (black dots) is shown. The strong destructive interference attests to an alignment close to the natural 2H configuration whereas constructive interference implies an alignment close to the 3R configuration, from which twist angles of $\theta = 53°$ (**a**), $\theta = 59°$ (**b**), and $\theta = 21°$ (**c**) can be deduced.

Supplementary Note 2

The experimental details of the ultrafast near-infrared (NIR) pump – mid-infrared (MIR) probe spectroscopy scheme are summarized in the Methods section of the manuscript. Supplementary Fig. 3 displays the NIR pump spectrum and the electric field of the MIR probe transient with its spectral amplitude and phase.

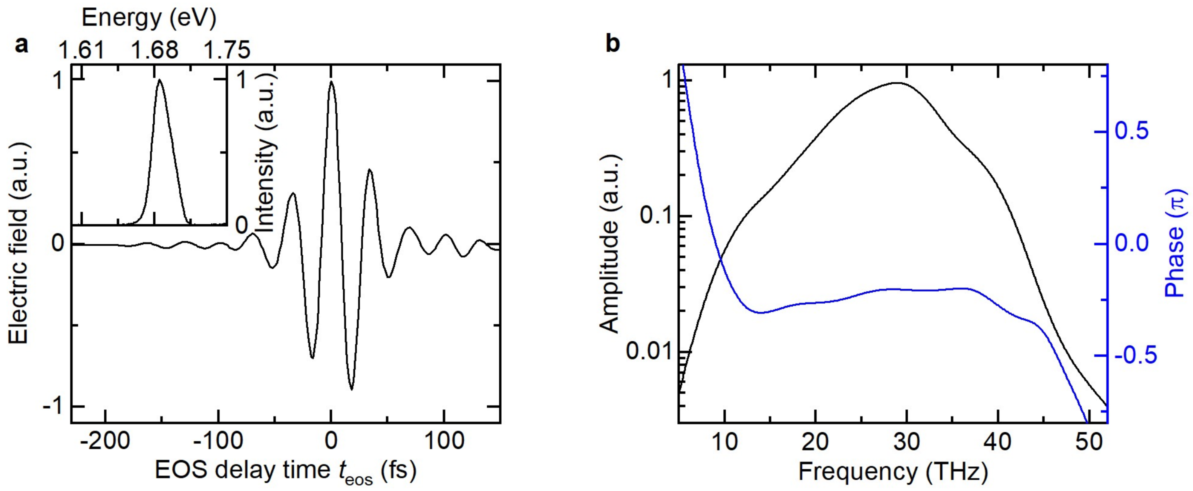

Supplementary Figure 3 | Ultrafast NIR pump-MIR probe spectroscopy. a, Electric field waveform of the MIR probe pulses as a function of the electro-optic delay time t_{eos}. Inset: Spectral intensity of the NIR pump pulses as a function of the photon energy. **b**, Spectral amplitude and phase of the MIR probe pulses as a function of frequency.

Supplementary Note 3

The twist-dependent delocalization of the excitons also manifests as an angle-dependent dynamical shift of the 1*s*–2*p* resonance. The dielectric responses of two BLs with $\theta = 60°$ and $\theta = 0°$ are shown in Supplementary Fig. 4a,b, for two representative pump-probe delay times ($t_{pp} = 0$ ps and 10 ps). For $t_{pp} = 0$ ps, a maximum in $\Delta\sigma_1$ at an energy of $\hbar\omega_{res} = 93$ meV arises for both samples. After 10 ps, clear deviations between the dielectric responses of the samples are discernible. For $\theta = 60°$, the resonance energy slightly blue shifts by ~7 meV, whereas for $\theta = 0°$ a redshift of ~30 meV occurs. Extracting the 1*s*–2*p* resonance energies of the dielectric function for a larger set of pump-probe delay times and twist angles yields the complete temporal evolution of the internal transitions, as depicted in Supplementary Fig. 4c. The resonance energy increases/decreases as a function of the pump-probe delay time for a twist angle of $\theta = 60°/\theta = 0°$ (gray/orange spheres). We attribute this blue/red shift to the formation of K-Λ excitons – subjected to different degrees of hybridization – after initial photoexcitation at the K valley. For $\theta = 60°$, the resulting K-Λ excitons feature a higher binding energy; the 1*s*–2*p* transition energy therefore increases as compared to the initially created K-K excitons owing to the larger electron effective mass in the Λ valley[2,3]. However, for $\theta = 0°$ the 1*s*–2*p* transition energy of the K-Λ excitons shows a clear red shift as compared to transition energy observed in $\theta = 60°$. Furthermore, for $\theta = 59°$, the evolution of the 1*s*–2*p* transition energy is similar to $\theta = 60°$, where the hybrid K-Λ exciton transition energy slightly increases due to the reduced interlayer hybridization. For intermediate twist angles of $\theta = 53°$, 30°, and 5°, the changes to the intraexcitonic resonance are less pronounced, but can be well explained by our microscopic theory.

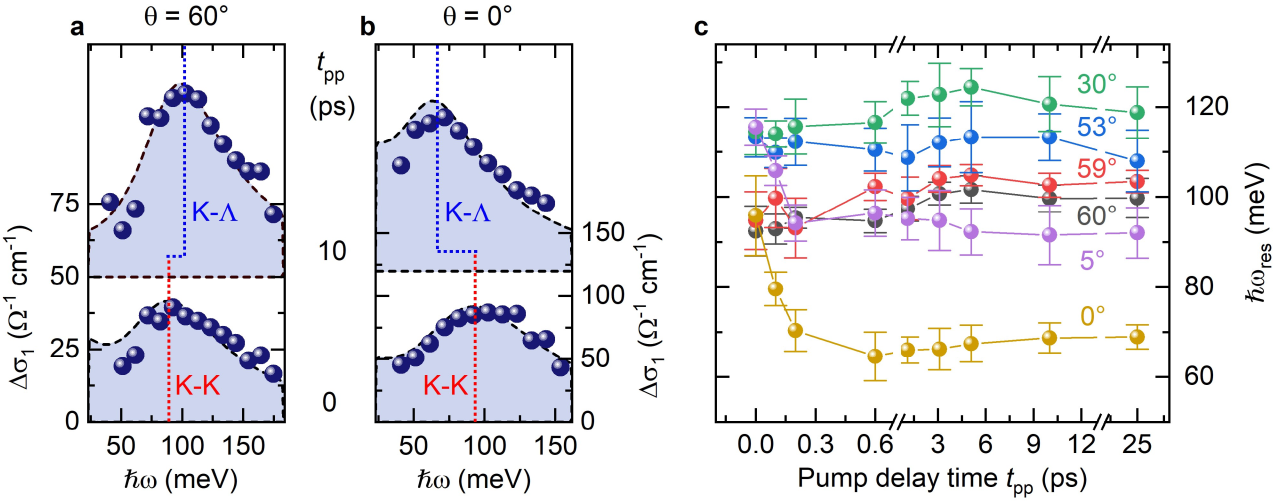

Supplementary Figure 4 | Ultrafast intervalley scattering. a,**b**, Pump-induced changes of the real parts of the optical conductivity $\Delta\sigma_1$ as a function of the photon energy, for two pump-probe delay times t_{pp} after resonant excitation of WSe_2 BLs with twist angles of $\theta = 60°$ (**a**) and $\theta = 0°$ (**b**). The dielectric responses (blue spheres) were recorded on the photoexcited WSe_2 BLs with a pump fluence $\Phi = 27$ μJcm^{-2}. The blue shaded areas represent the fits to those data with a phenomenological model. The red/blue dashed line indicates the 1*s*–2*p* resonance of the K-K and K-Λ exciton, respectively. **c**, 1*s*–2*p* resonance energy $\hbar\omega_{res}$ as a function of the pump-probe delay time t_{pp} for different twist angles θ. The samples were kept at a temperature of 5 K. The error bars represent the 95% confidence interval of the fitting procedure.

Supplementary Note 4

To investigate the density-dependent renormalization of the hybrid exciton binding energy, we recorded the dielectric response function of WSe_2 BLs with twist angles of $\theta = 60°$, 5°, and 0° at different pump fluences using a fixed pump-probe delay time of $t_{pp} = 5.1$ ps (Supplementary Fig. 5). For all samples and pump fluences, we observe a clear maximum in the real part of the optical conductivity $\Delta\sigma_1$ and a corresponding dispersive feature in the real part of the dielectric function $\Delta\varepsilon_1$, arising from the resonant response of the intraexcitonic 1*s*–2*p* transition.

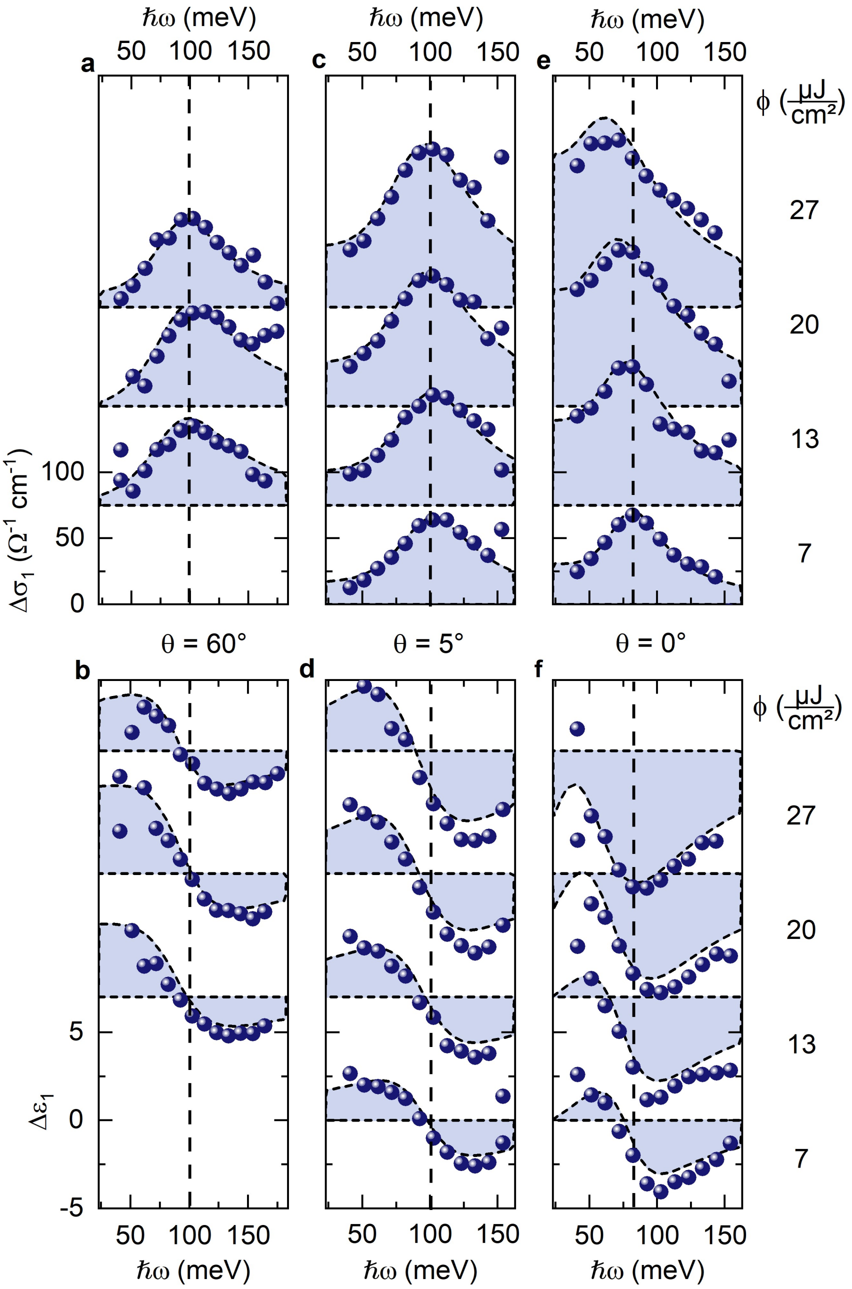

Supplementary Figure 5 | Fluence dependence of the binding energy of K-Λ hybrid excitons. a-f, Pump-induced changes of the real parts of the optical conductivity $\Delta\sigma_1$ (**a**, **c**, **e**) and real parts of the dielectric function $\Delta\varepsilon_1$ (**b**, **d**, **f**) as a function of the photon energy, for different pump fluences Φ. The data were recorded at a pump-probe delay time of $t_{pp} = 5.1$ ps after resonant excitation for three different WSe_2 BLs with twist angles of $\theta = 60°$, $\theta = 5°$, and $\theta = 0°$. For $\theta = 60°$, an extraction of the dielectric function was not feasible for $\Phi = 7$ μJcm^{-2}. The blue spheres indicate the experimental data and the blue shaded areas represent the fits with a phenomenological model. The samples were kept at a temperature of 5 K.

The characteristic spectral features of the pristine BL and the sample with a twist angle of $\theta = 5°$ remain at an energy of ~100 meV for all pump fluences. For $\theta = 60°$, no significant pump-probe signal was observed for the lowest pump fluence of $\Phi = 7$ μJcm^{-2}. Employing our phenomenological model to fit the experimental spectra (see Methods), we quantify the renormalization of the 1*s*–2*p* resonance energy $\hbar\omega_{res}$ (Fig. 4c) as a function of the pump fluence Φ. While $\hbar\omega_{res}$ of the BL with 2H stacking and the one with $\theta = 5°$ are hardly affected and remain nearly unchanged at ~100 meV, the pump fluence has a dramatic impact for $\theta = 0°$. Here the 1*s*–2*p* resonance energy drops from $\hbar\omega_{res} = 83$ meV to 63 meV as the pump fluence is increased from 7 μJcm^{-2} to 27 μJcm^{-2}. These observations are in line with our microscopic understanding of the twist angle-dependent hybridization of excitonic states as discussed in the main text.

Supplementary Note 5

Here, we present the theoretical approach used to determine eigenenergies and wavefunctions of the energetically lowest lying excitonic states in the BL. Since K-Λ excitons are known to be the energetically lowest states in the WSe_2 BL(ref. [2]), we start with a BL Hamilton operator restricted to electrons and holes at these high symmetry points. Here, the electronic contribution reads

$$H = \sum_{i\mathbf{k}} (\varepsilon^{c}_{i\mathbf{k}} c^{\dagger}_{i\mathbf{k}} c_{i\mathbf{k}} + \varepsilon^{v}_{i\mathbf{k}} v^{\dagger}_{i\mathbf{k}} v_{i\mathbf{k}}) + \sum_{ij\mathbf{k}\mathbf{k}'\mathbf{q}} W^{ij}_{\mathbf{q}}\, c^{\dagger}_{i\mathbf{k}+\mathbf{q}} v^{\dagger}_{j\mathbf{k}'-\mathbf{q}} v_{j\mathbf{k}'} c_{i\mathbf{k}} + \sum_{i\neq j,\mathbf{k}} h^{ij}_{\mathbf{q}}\, c^{\dagger}_{i\mathbf{k}+\mathbf{q}} c_{j\mathbf{k}}\,, \quad (1)$$

with annihilation (creation) operators of conduction $c^{(\dagger)}_{i\mathbf{k}}$ and valence $v^{(\dagger)}_{i\mathbf{k}}$ band electrons with the momentum **k** and in the layer $i = 1,2$. Here, the eigenenergies of the layer-localized basis functions are given by the effective mass approximation $\varepsilon^{\lambda}_{i\mathbf{k}} = \varepsilon^{\lambda}_{i0} \pm \hbar^2 k^2/(2m^{\lambda}_i)$ with effective masses m^{λ}_i of the Λ valley electrons and K valley holes. Effective masses, bandgaps and energetic separations of valleys entering $\varepsilon^{\lambda}_{i0}$ are taken from DFT calculations[3]. The second term of Eq. 1 contains the Coulomb interaction between electrons and holes with the matrix elements $W^{ij}_{\mathbf{q}}$. The latter are derived in a semiclassical approach assuming anisotropic homogeneous slabs to account for the dielectric environment created by the transition metal dichalcogenide (TMD) BL system[3]. Here, we use *ab initio* parameters[4] for the dielectric tensor of WSe_2 and $\epsilon_{\text{diam}} = 5.8$ (ref. [5]). The last term of Eq. 1 reflects the overlap of electronic wavefunctions of the two layers giving rise to interlayer hybridization. We focus on the hybridization of electrons at the Λ valley, which is known to be orders of magnitude stronger than at the K valley[6]. The interlayer hopping is momentum conserving, which in the valley-local momentum coordinates requires a momentum transfer $h^{ij}_{\mathbf{q}} = h_{\Lambda}\delta_{\mathbf{q},\Lambda_j-\Lambda_i}$ (ref. [7]) depending on the alignment of the layer Brillouin zones and thus on the stacking angle. The interlayer hopping strength at the Λ-point, h_{Λ} is extracted from DFT calculations of the BL bandstructure (cf. section 6) and depends on the interlayer distance and therefore also on the twist angle.

To diagonalize the above Hamiltonian, we first transform the Hamiltonian into an exciton frame to account for the Coulomb interaction on a Hartree-Fock level using excitonic operators $X^{ij\,\dagger}_{\mu\mathbf{Q}} = \sum_{\mathbf{k}} \psi^{ij}_{\mu}(\mathbf{k})\; c^{\dagger}_{i,\mathbf{k}+\alpha_{ij}\mathbf{Q}} v_{j,\mathbf{k}-\beta_{ij}\mathbf{Q}}$ (ref. [4,8]) with mass factors $\alpha_{ij}(\beta_{ij}) = m^{c}_i(m^{v}_j)/m^{c}_i + m^{v}_j$, center-of-mass momentum **Q** and the wavefunction $\psi^{ij}_{\mu}(\mathbf{k})$ for intra- $(i = j)$ and interlayer $(i \neq j)$ excitons. The latter fulfill the BL Wannier equation[8]. In the exciton picture, the Hamiltonian reads

$$H = \sum_{ij\mu\mathbf{Q}} E^{ij}_{\mu\mathbf{Q}} X^{ij\,\dagger}_{\mu\mathbf{Q}} X^{ij}_{\mu\mathbf{Q}} + \sum_{i\neq j,n\nu\mu\mathbf{Q}} T^{in,jn}_{\nu\mu}\, X^{in\,\dagger}_{\nu\mathbf{Q}+\Lambda_j-\Lambda_i} X^{jn}_{\mu\mathbf{Q}} \quad (2)$$

with the excitonic interlayer hopping matrix $T^{in,jn}_{\nu\mu} = h_{\Lambda}F^{in,jn}_{\nu\mu} = h_{\Lambda}\sum_{\mathbf{k}} \psi^{jn}_{\mu}(\mathbf{k})^{*}\, \psi^{in}_{\nu}(\mathbf{k} + \beta_{in}[\Lambda_j - \Lambda_i])$ (ref. [6]) and exciton eigenenergies $E^{ij}_{\mu\mathbf{Q}} = E^{ij}_{\mu 0} + \varepsilon^{c}_{i0} - \varepsilon^{v}_{j0} + \hbar^2 Q^2/(2[m^{c}_i + m^{v}_j])$. Finally, we diagonalize the exciton Hamiltonian yielding $H = \sum_{\gamma\mathbf{Q}} \tilde{E}_{\gamma\mathbf{Q}}\, Y^{\dagger}_{\gamma\mathbf{Q}} Y_{\gamma\mathbf{Q}}$, where we have introduced exciton-hybrids $Y^{\dagger}_{\gamma\mathbf{Q}} = \sum_{ij\mu} C_{\gamma\mathbf{Q}}(i,j,\mu)\, X^{ij\,\dagger}_{\mu\mathbf{Q}-\Lambda_i}$, resulting from the hybridization eigenproblem $E^{ij}_{\mu\mathbf{Q}-\Lambda_i} C_{\gamma\mathbf{Q}}(i,j,\mu) + \sum_{n\neq i,\nu} T^{ij,nj}_{\mu\nu} C_{\gamma\mathbf{Q}}(n,j,\nu) = \tilde{E}_{\gamma\mathbf{Q}} C_{\gamma\mathbf{Q}}(i,j,\mu)$. The coefficients $C_{\gamma\mathbf{Q}}(i,j,\mu)$ determine how strongly the exciton state (i,j,μ) is contributing to the hybrid exciton with quantum numbers $(\gamma,\mathbf{Q})$. For the numerical calculation of the hybrid states we take into account the 56 energetically lowest exciton eigenstates for both intra- and interlayer excitons including angular momenta of up to $l = 3$ (*s*-, *p*-, *d*- and *f*-type states).

The observed stacking dependence of the hybridization can be qualitatively understood by simplifying the theoretical approach. For twist-angles $\theta \neq 0°$, the mixing of intra- and interlayer excitons with different quantum numbers μ (e.g. mixing of 2*s* and 2*p*) can significantly contribute to the hybrid states (in particular for the excited states). In contrast, for $\theta \approx 0°$ the exciton form factor for states with different quantum numbers becomes very small, i.e. $F_{\nu\mu}^{in,jn} \approx F_{\mu}^{in,jn}\delta_{\nu\mu}$ so that the hybridization eigenproblem can be solved analytically yielding the simple form

$$E_{\mu\mathrm{Q}}^{\mathrm{hyb},\pm} = \frac{1}{2}\left(E_{\mu\mathrm{Q}}^{\mathrm{inter}} + E_{\mu\mathrm{Q}}^{\mathrm{intra}}\right) \pm \frac{1}{2}\sqrt{\left(E_{\mu\mathrm{Q}}^{\mathrm{inter}} - E_{\mu\mathrm{Q}}^{\mathrm{intra}}\right)^2 + 4\left|T_{\mu}\right|^2}\,. \qquad (3)$$

This resembles the well-known anti-crossing behavior of hybridizing particles. Furthermore, for all configurations, the lowest hybrid exciton is mostly composed of intralayer states. Therefore, the energy reduction $\delta_{\mu\mathrm{Q}}^{\mathrm{hyb}} = E_{\mu\mathrm{Q}}^{\mathrm{intra}} - E_{\mu\mathrm{Q}}^{\mathrm{hyb},-}$ of the intralayer exciton due to delocalization (measure for the degree of hybridization) is given by $\delta_{\mu\mathrm{Q}}^{\mathrm{hyb}} = -\frac{\Delta_{\mu\mathrm{Q}}}{2} + \frac{1}{2}\sqrt{\Delta_{\mu\mathrm{Q}}^2 + 4\left|T_{\mu}\right|^2}$, with the detuning of inter- and intralayer states $\Delta_{\mu\mathrm{Q}} = E_{\mu\mathrm{Q}}^{\mathrm{inter}} - E_{\mu\mathrm{Q}}^{\mathrm{intra}} > 0$. We find that $\delta^{\mathrm{hyb}} > 0$ and $\partial\delta^{\mathrm{hyb}}/\partial\Delta < 0$. Therefore, the red shift reduces monotonously, meaning that the hybridization becomes weaker with increasing detuning of the mixing states. This explains the different hybridization of states at $\theta = 0°$ and 60° stacking, since the layer inverted spin-orbit coupling leads to a large separation of intra- and interlayer states at 60°. In contrast, the mixing electronic bands are degenerate at 0°. For $\theta \neq 0°$ or 60°, the dispersion of intra- and interlayer excitons becomes misaligned in momentum, leading to a **Q**-dependent detuning (Supplementary Fig. 6). This already leads to an upshift of the hybrid dispersion (violet lines), since the interlayer state (orange line) hybridizing with the minimum of the intralayer dispersion (blue line) is moving upwards in energy and the relevant detuning increases.

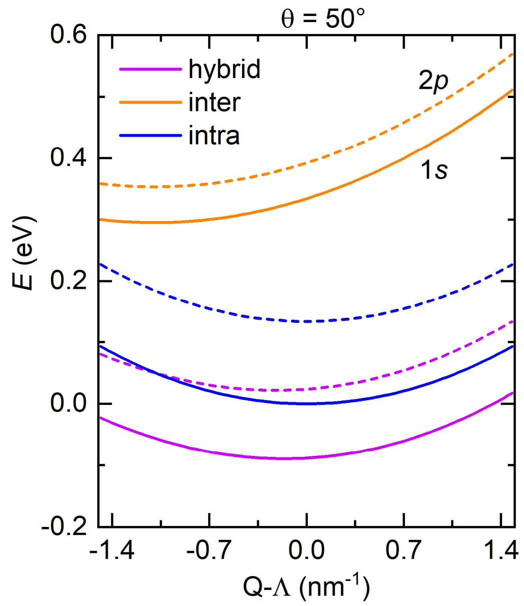

Supplementary Figure 6 | Exciton dispersion relation for $\theta = 50°$. Exciton dispersion relations as a function of the center-of-mass momentum Q for the 1*s* (solid lines) and 2*p* (dashed lines) state of the inter/intralayer and hybrid exciton.

There are two more effects leading to a reduced exciton hybridization in twisted BLs: (i) As a result of stacking dependent interlayer distances, the electronic hopping integral h_{Λ} decreases for twisted samples[7,9] (cf. section 6). (ii) The excitonic hopping integral $T_{\mu}(\mathrm{d}\Lambda) \propto F_{\mu}(\mathrm{d}\Lambda) = \sum_{\mathbf{k}} \psi_{\mu}^{\mathrm{inter}}(\mathbf{k})^* \psi_{\mu}^{\mathrm{intra}}(\mathbf{k} + \beta\mathrm{d}\Lambda)$ decreases with increasing momentum mismatch $|\mathrm{d}\Lambda| = |\Lambda_1 - \Lambda_2| \sim \theta$. This reflects the decreasing in-plane overlap of intra- and interlayer exciton wavefunctions. The

phenomenon (ii) explains why the $1s$-$2p$ transition energy increases when hybridization decreases. The large $2p$ state orbitals correspond to narrower momentum space wavefunctions compared to the $1s$ ground state (see Fig. 3c of the main manuscript). Therefore, the in-plane overlap F_μ decreases faster with the twist angle for the excited states than for the ground states, i.e. $|\partial T_{1s}/\partial\theta| < |\partial T_{2p}/\partial\theta|$. Hence, the $1s$-$2p$ transition energy increases, since the $2p$ state blue-shifts with a larger rate than the $1s$ state.

Higher-order corrections to the energy levels of excitons are expected to have a negligible effect on our current theoretical analysis, for the following reasons:
(i) The expected splitting between the p_+ and p_- states lies in the range of 10 – 25 meV for K-K excitons in monolayer TMDs in vacuum as derived in the literature[10–12]. Recently, this splitting was also confirmed experimentally[13] and an energy difference of ~14 meV was reported, in line with the theoretical predictions. Owing to the enhanced dielectric screening experienced by excitons in a bilayer TMD on a diamond substrate, we expect that the corresponding Berry curvature-induced splitting should be even smaller. Specifically, exciton binding energies on the order of 200 meV suggest energy differences between $2p_+$ and $2p_-$ states within the range of only 5 – 13 meV.
(ii) The considerations so far have neglected the fact that the electrons of the energetically favourable excitons in WSe_2 reside in the Λ-valley, where the Berry curvature is significantly smaller[14] than in the K-valley. Hence, we expect that the energetic difference between the $2p_+$ and $2p_-$ states of the hybrid excitons in WSe_2 homobilayers should lie well below 10 meV.
On the experimental side, we cannot currently resolve such higher-order corrections. First, the mid-infrared probe pulses are linearly polarized for the extraction of the pump-induced dielectric response of the homobilayers. Since linearly polarized light is a superposition of right- and left-circularly polarized light, we simultaneously drive 1s–$2p_+$ and 1s–$2p_-$ transitions. With this approach, we effectively obtain an averaged $1s$–$2p$ transition energy. Second, the experimental line width of the internal excitonic transitions amounts to tens of meV, which obscures the inhomogeneous broadening induced by the p_+/p_- splitting.
Therefore, even without Berry curvature-induced corrections to the excitonic energy levels, our effective-mass model already effectively captures the essential mechanism responsible for the renormalization of the exciton binding energy with twist angle: the hybridization of intra- and interlayer excitons.

Calculating the energy levels of hybrid excitons in WSe_2 homobilayers at twist angles close to $\theta = 30°$ is not feasible at this moment because the interlayer hopping cannot be extracted from the single-particle bandstructure in this case. Consequently, we have decided to limit our theoretical consideration to small misalignments from 0° and 60° for the following reasons:
The calculation of interlayer-intervalley excitons in twisted TMDs based solely on DFT is extremely challenging and has so far not been demonstrated yet. Instead, we use a combination of DFT and an effective-mass Wannier model to predict intervalley exciton energies in twisted homobilayers. In perfectly aligned bilayers, the initially degenerate bands of the constituent monolayers are split depending on the strength of the interlayer hopping. For twisted bilayers, this does, not hold because zone folding, for example, gives rise to a mixing of multiple bands. Consequently the band splitting is no longer a good measure for the interlayer hopping. Therefore, we use the DFT bandstructure of perfectly aligned bilayers and assume that the hopping term extracted from the band splitting to a first approximation only changes slowly with twist angle in the vicinity of a given high symmetry point and can therefore also be used for slightly misaligned samples. This approximation is, however, limited to small twist angles. At large twist angles such as 30°, the hybridization between different high-symmetry points such as Λ of one layer and Σ (which lies between Λ and Λ') of the other layer will strongly affect the interlayer hopping.

Supplementary Note 6

The electronic band structures of the WSe_2 BLs were simulated on the basis of density functional theory in the Perdew-Burke-Ernzerhof approximation, as implemented in the Quantum ESPRESSO package[15]. Integrations in reciprocal space were performed on a Monkhorst-Pack grid of 12x12x1 points in the Brillouin zone. The core electrons were replaced by normconserving pseudopotentials from the Pseudo Dojo library[16], where the s and p semi-core electrons of tungsten and the semicore d electrons of selenium were included in the set of valence electrons. We used a cutoff energy of 90 Ry (1224 eV) for the employed planewave basis set, ensuring well converged ground state electron densities and wavefunctions. Using these parameters, we optimized the atomic positions and in-plane lattice constants of twisted WSe_2 BLs with different relative twist angles, such as 60° (corresponding to the 2H-stacking order in bulk WSe_2) and 46.8°. We used the resulting atomic geometries to derive an average equilibrium distance between the two tungsten sublayers. We included semi-empirical van der Waals corrections from the PBE+D3 (ref. [17]) scheme for these calculations, which yield excellent predictions of the in- and out-of-plane lattice constants of layered systems[18,19].

Based on the optimized geometry of 2H-stacked BL WSe_2 with an optimized interlayer distance of 6.5 Å, we subsequently simulated the evolution of the electronic bandstructure (neglecting spin-orbit-coupling effects) with the interlayer interaction by rigidly shifting the upper layer in out-of-plane direction, thus varying the interlayer distance in the range between 6.5 Å and 7.25 Å. The interlayer hopping parameter h_Λ introduced in section 5 is extracted from the BL bandstructure without spin-orbit interaction. In particular, the interlayer hybridization gives rise to a $\mathbf{k}$-dependent splitting of the electronic bands $\Delta E_{\mathbf{k}}$, allowing to determine the hopping integral $h_\Lambda = \Delta E_\Lambda/2$ (ref. [6]). We find e.g. $h_\Lambda \approx 230$ meV for the interlayer distance of 6.5 Å ($\theta = 60°$) and an almost linear dependence on interlayer distance, giving rise to a reduced hopping of about $h_\Lambda \approx 200$ meV at 6.75 Å ($\theta = 46.8°$) as the result of a reduced orbital overlap. To obtain a continuous twist angle dependence of the hopping parameter, the interlayer distances obtained for the computationally feasible angles are interpolated linearly for the intermediate angles.

To obtain the full bandstructure for 2H-stacked WSe_2, we combined spin-orbit coupling and quasiparticle energy corrections from the G_0W_0 approximation. Here, we first used the YAMBO code[20] to calculate the quasiparticle corrections with SOI effects on a regular 9x9 k-point grid. The dynamic dielectric function was represented on a discrete 9×9 q-point grid of transferred momenta in reciprocal space and by the Godby-Needs plasmon-pole approximation in frequency space. Local field effects and exchange interactions were included up to a cutoff energy of 300 eV and 650 eV, respectively. We truncated the Coulomb interaction in the non-periodic direction through a Wigner-Seitz cutoff scheme[18]. The correct asymptotic behavior of the dielectric screening for transferred momenta close to the Γ point was imposed through an isotropic model dielectric function that was used previously to great success[18,21]. While it is sufficient to capture the correct quasiparticle band dispersion, it causes an overestimation of the electronic band gap sizes. We thus performed additional GW calculations (neglecting spin-orbit interactions) with the BerkeleyGW code[22], where we used the recently proposed Nonuniform Neck Subsampling method[23] to derive an additional scissor shift correction for the electronic band gaps. The electronic band structures were then derived from the corrected electronic bands through Wannier interpolation.

Supplementary References

1. Lin, K.-Q., Bange, S. & Lupton, J. M. Quantum interference in second-harmonic generation from monolayer WSe_2. *Nat. Phys.* **15**, 242–246 (2019).

2. Deilmann, T. & Thygesen, K. S. Finite-momentum exciton landscape in mono- and bilayer transition metal dichalcogenides. *2D Mater.* **6**, 035003 (2019).

3. Kormányos, A. *et al.* k.p theory for two-dimensional transition metal dichalcogenide semiconductors. *2D Mater.* **2**, 049501 (2015).

4. Katsch, F., Selig, M., Carmele, A. & Knorr, A. Theory of Exciton–Exciton Interactions in Monolayer Transition Metal Dichalcogenides. *Phys. status solidi* **255**, 1800185 (2018).

5. Laturia, A., Van de Put, M. L. & Vandenberghe, W. G. Dielectric properties of hexagonal boron nitride and transition metal dichalcogenides: from monolayer to bulk. *npj 2D Mater. Appl.* **2**, 6 (2018).

6. Ruiz-Tijerina, D. A. & Fal'ko, V. I. Interlayer hybridization and moiré superlattice minibands for electrons and excitons in heterobilayers of transition-metal dichalcogenides. *Phys. Rev. B* **99**, 125424 (2019).

7. Wang, Y., Wang, Z., Yao, W., Liu, G.-B. & Yu, H. Interlayer coupling in commensurate and incommensurate bilayer structures of transition-metal dichalcogenides. *Phys. Rev. B* **95**, 115429 (2017).

8. Ovesen, S. *et al.* Interlayer exciton dynamics in van der Waals heterostructures. *Commun. Phys.* **2**, 23 (2019).

9. Yeh, P.-C. *et al.* Direct Measurement of the Tunable Electronic Structure of Bilayer MoS_2 by Interlayer Twist. *Nano Lett.* **16**, 953–959 (2016).

10. Zhou, J., Shan, W.-Y., Yao, W. & Xiao, D. Berry Phase Modification to the Energy Spectrum of Excitons. *Phys. Rev. Lett.* **115**, 166803 (2015).

11. Srivastava, A. & Imamoğlu, A. Signatures of Bloch-Band Geometry on Excitons: Nonhydrogenic Spectra in Transition-Metal Dichalcogenides. *Phys. Rev. Lett.* **115**, 166802 (2015).

12. Glazov, M. M. *et al.* Intrinsic exciton-state mixing and nonlinear optical properties in transition metal dichalcogenide monolayers. *Phys. Rev. B* **95**, 035311 (2017).

13. Yong, C.-K. *et al.* Valley-dependent exciton fine structure and Autler–Townes doublets from Berry phases in monolayer $MoSe_2$. *Nat. Mater.* **18**, 1065–1070 (2019).

14. Kormányos, A., Zólyomi, V., Fal'ko, V. I. & Burkard, G. Tunable Berry curvature and valley and spin Hall effect in bilayer MoS_2. *Phys. Rev. B* **98**, 035408 (2018).

15. Giannozzi, P. *et al.* QUANTUM ESPRESSO: a modular and open-source software project for quantum simulations of materials. *J. Phys. Condens. Matter* **21**, 395502 (2009).

16. van Setten, M. J. *et al.* The PseudoDojo: Training and grading a 85 element optimized norm-conserving pseudopotential table. *Comput. Phys. Commun.* **226**, 39–54 (2018).

17. Grimme, S., Ehrlich, S. & Goerigk, L. Effect of the damping function in dispersion corrected density functional theory. *J. Comput. Chem.* **32**, 1456–1465 (2011).

18. Gillen, R. & Maultzsch, J. Interlayer excitons in $MoSe_2/WSe_2$ heterostructures from first principles. *Phys. Rev. B* **97**, 165306 (2018).

19. Tornatzky, H., Gillen, R., Uchiyama, H. & Maultzsch, J. Phonon dispersion in MoS_2. *Phys. Rev. B* **99**, 144309 (2019).

20. Marini, A., Hogan, C., Grüning, M. & Varsano, D. yambo: An ab initio tool for excited state calculations. *Comput. Phys. Commun.* **180**, 1392–1403 (2009).

21. Ismail-Beigi, S. Truncation of periodic image interactions for confined systems. *Phys. Rev. B* **73**, 233103 (2006).

22. Deslippe, J. *et al.* BerkeleyGW: A massively parallel computer package for the calculation of the quasiparticle and optical properties of materials and nanostructures. *Comput. Phys. Commun.* **183**, 1269–1289 (2012).

23. da Jornada, F. H., Qiu, D. Y. & Louie, S. G. Nonuniform sampling schemes of the Brillouin zone for many-electron perturbation-theory calculations in reduced dimensionality. *Phys. Rev. B* **95**, 035109 (2017).
